# The use of amplitude-integrated electroencephalography combined with continuous conventional electroencephalography during therapeutic hypothermia for an infant with postnatal cardiac arrest

**DOI:** 10.1186/2193-1801-3-373

**Published:** 2014-07-21

**Authors:** Asuka Ito, Yasunori Mishima, Yukari Koga, Mayu Saho, Teruyuki Hiraki, Kazuo Ushijima

**Affiliations:** Department of Anesthesiology, Kurume University School of Medicine, 67 Asahi-machi, Kurume, Fukuoka, 830-0011 Japan

**Keywords:** Amplitude-integrated electroencephalography, Continuous conventional electroencephalography, Therapeutic hypothermia, Cardiac arrest, Hypoxic ischemic encephalopathy, Seizures

## Abstract

**Introduction:**

Amplitude-integrated electroencephalography (aEEG) has been employed in therapeutic hypothermia (TH) trials of neonates after perinatal hypoxic-ischemic encephalopathy (HIE). We present a case report involving the use of aEEG during TH with continuous conventional electroencephalography (cEEG) for an infant who experienced postnatal intraoperative cardiac arrest.

**Case description:**

A five-month-old infant developed cardiac arrest during operation. Return of spontaneous circulation was achieved after one hour of cardiopulmonary resuscitation. Therapeutic hypothermia was applied with neuromuscular blockades. During the TH, the brain function and seizures were monitored with aEEG, which can also display continuous cEEG. Intermittent and discrete seizures were detected on aEEG and confirmed with raw cEEG during the TH and rewarming periods. Several kinds of antiepileptic drugs (AEDs) were administered to manage seizures according to the findings of aEEG with cEEG. Seizures were controlled by the treatments, and she showed no clinical seizures after TH and AED discontinuation.

**Discussion and evaluation, conclusions:**

This case indicated the possibility that the use of aEEG with continuous cEEG for a postnatal infant after cardiac arrest was feasible to detect and assess seizures and the effects of antiepileptic therapy while undergoing TH.

## Background

Patients with cardiac arrest show high rates of mortality and neurologic morbidity. Therapeutic hypothermia (TH) has been demonstrated to improve the neurological outcome in adults after cardiac arrest, and is now considered the standard of care (Bernard et al. 2002). Based on extrapolation from evidence of existing nonpediatric studies, TH is recommended for children who remain comatose following resuscitation from cardiac arrest (Kleinman et al. 2010). Hypoxic ischemic encephalopathy (HIE) after cardiac arrest may cause seizures, which are associated with a poor outcome (Young et al. 1996). The early detection and appropriate treament of seizures are necessary to improve the neurologic outcome. Conventional electroencephalography (cEEG) is the standard method to confirm seizures. Amplitude-integrated electroencephalography (aEEG) has been introduced for seizure detection in neonates with perinatal asphyxia (van Rooij et al. 2005). This case report presents the use of aEEG combined with continuous cEEG during TH for a five-month-old infant with postnatal cardiac arrest.

## Case description

**Figure 1:**
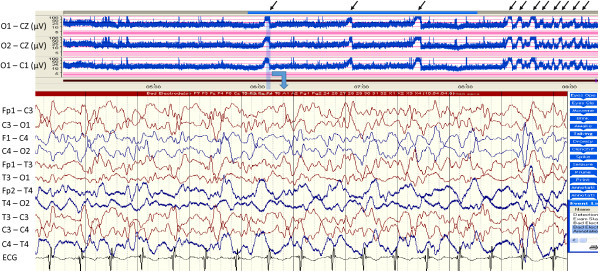
**The waveforms of aEEG and cEEG for 70 hours after the induction of TH.** The upper panel represent amplitude-integrated EEG (aEEG), showing a repetitive narrowing bandwidth with a sharp rise in the lower margin accompanied by a smaller rise in the upper margin (arrows), which was suspected as intermittent and discrete seizrues. Raw cEEG tracing corresponding to light blue band in aEEG confirms the waveform of aEEG as seisures.

A 2,540-g female infant was delivered spontaneously at 37 weeks of gestation with esophageal atresia and a distal thoracoesophageal fistula (TEF). A primary anastomosis of the esophagus and ligation of the fistula was performed under general anesthesia at the age of 1 day. However, recurrent TEF was found with her. She underwent gastrostomy at the age of 42 days and was decided to perform re-operation and closure of TEF at the age of 5 months. During the operation, cardiac arrest occurred and cardiopulmonary resuscitation was initiated. Return of spontaneous circulation was achieved after one hour, and she was admitted to a pediatric intensive care unit (ICU). Immediately, continuous assessments of the brain function and seizure detection were performed using aEEG (Nicolet One® System, CareFusion, San Diego, CA, USA), which can also monitor continuous cEEG. The infant underwent cEEG with 11 electrodes applied by using the 10 - 20 international system, at the following locations: Fp1, Fp2, T3, T4, C3, Cz, C4, O2 and O1 to record EEG activity from frontal, temporal, central and occipital areas. The 1-channel aEEG traces those were obtained as the voltage potential difference between the O1 - O2, O1 - Cz, and O2 - Cz were continuously monitored. A severely supressed background was found on aEEG and TH was induced with whole-body cooling using a cooling mattress for neuroprotection. Her core body temperature was monitored continuously at the rectum. The target core temperature of 33.5°C was achieved in one hour. While cooling, she was mechanically ventilated and administered muscle relaxant and fentanyl. By five hours after the induction of TH, disorganized, low amplitude delta activities appeared on cEEG and amplitude of 5 to 50μV with weak cycling appeared on aEEG. Seventy hours after the induction of TH, an abrupt, sharp rise in the lower margin accompanied by a smaller rise in the upper margin with narrowing of the band width were repetitively detected on aEEG (arrows in Figure 1) with an amplitude of 5 to 50μV, and the corresponding raw cEEG confirmed to be seizures (Figure 1). Phenobarbital was administered, and the subsequent aEEG revealed an apparent decrease in seizures. Eighty-four hours after the induction of hypothermia, the patient was rewarmed and the rectal temperature was allowed to rise by no more than 0.5°C per hour to a maximum of 37°C. During the rewarming period, four episodes of seizures on both aEEG and cEEG were detected. They were effectively treated with midazolam followed by phenytoin, but the interval between seizures shortened, suggesting highly refractory epileptic activity. No electrographical seizures were detected on aEEG for several days after starting the continuous administration of thiamylal sodium with its dose of 3 mg per kg per hour. The aEEG monitoring was discontinued after thiamylal sodium was tapered. She was then extubated without respiratory distress, and showed no clinical seizures thereafter. Auditory brainstem responses identified no apparent abnormality based on wave amplitudes on postoperative day (POD) 34. Cerebral magnetic resonance imaging on POD 36 showed diffuse high intensity areas on T2-weighed image in her bilateral frontal and occipital lobes and putamen, which suspected hypoxic brain damage. She was alive with a cerebral outcome of Cerebral Performance Category of 3, which means severe cerebral disability, dependent on others for daily support because of impaired brain function at 6-month follow up.

## Discussion and conclusion

Neurological care for patients who are successfully resuscitated with post cardiac arrest syndrome (PCAS) remains challenging. Patients with PCAS can be occurred with seizures in 15 to 44% of frequency (Khot and Tirschwell 2006). They are frequently nonconvulsive, difficult to control, and associated with higher rates of neurological morbidity and mortality (Krumholtz et al. 1998). Nonconvulsive seizures are electrographic seizures with little or no overt clinical manifestations, which may not be recognized without continuous monitoring. Moreover, the diagnosis of seizures is difficult in clinically ill infants who are intubated, sedated, or administered neuromuscular blockades, such as our case. Therefore, continuous cEEG monitoring is required for prompt and reliable nonconvulsive seizure detection.

The interpretation of cEEG requires an encephalographer with specific training in cEEG. However, in most units, technicians and experienced clinical neurophysiologists are not available 24 hours a day. To improve these limitations of cEEG, a simpler methodology for monitoring the cerebral function has been developed. Over the past decade, aEEG has become a bedside cerebral function monitoring method in the neonatal ICU, especially to monitor the neurologic status, seizures, and effects of therapies, and to predict the neurological outcome following HIE (Spitzmiller et al. 2007). The aEEG tracing is viewed with a highly compressed time scale, at a slow rate of 6 cm/hr, compared to 3 cm/sec of cEEG. With its time-compressed output, aEEG is able to provide a simpler means of following cerebral function trends in the ICU without the need for experienced technicians for the application and interpretation of the analog cEEG system.

The aEEG represents the processed and compressed cEEG signal from one or two channels. The advantages of the simplified electrode system are that it is faster to apply and easier to maintain. However, artifacts those influence the voltage and width of the aEEG band could make aEEG problematic as an assessment tool. Artifacts commonly mistaken for seizrues on aEEG include gross movemet of the subject, muscle activity, and electrical interference (Rosen. 2006). Reviewing the corresponding raw EEG tracing is necessary to confirm seizures seen on aEEG. The aEEG that we used was able to display both tracings of aEEG and cEEG in real time simultaneously. Moreover, the equipment allowed the interpreter to intermittently call up and display the raw cEEG corresponding to a suspicious event observed on the compressed aEEG trace. This may improve the accuracy of aEEG-based diagnosis for seizures in our case.

Seizures appeared at seventy hours during hypothermia and at eighty-four hours during rewarming after the induction of TH in our patient. The duration of treatment with hypothermia has varied in experimental studies. Studies with birth asphyxia showed that TH up to 72 hours after resuscitation has an acceptable safety profile (Gluckman et al. 2005; Shankaran et al. 2005). Several complications associated with TH are impaired coagulation, increased bleeding, impaired immune system and increased infection rates (Sessler 2001). We decided to continue rewarming with using antiepileptic drugs (AEDs) to control seizures in 84 hours after induction of hypothermia because these side effects of TH could affect postoperative condition.

A number of studies have suggested that early intervention for seizures is associated with an effective response to anticonvulsant treatment and a better outcome (Eriksson et al. 2005), although an evidence-based therapeutic protocol for seizures has not yet been developed. However, there is also concern about possible adverse effects of AEDs on the developing brain (Glass and Wirrell 2009; Dzhala et al. 2010) in addition to the potential harm caused by seizures to the immature brain (Glass et al. 2009). The change of AEDs for seizures according to the observation of aEEG in our coma case may be valuable to achieve a better balance between the efficacy of AEDs for seizures and their potential neurotoxicity in the immature brain. The application of aEEG with continuous cEEG could help evaluate the effect of AEDs and avoid their excessive and continuously ineffective use. Based on this single-case experience, we conclude that the use of aEEG with continuous cEEG in infants after cardiac arrest may facilitate assessing the brain function and effect of anticonvulsant therapy during TH.

## Consent

Informed consent was obtained from the patient’s parent for the publication of this report and any accompanying images.

## References

[CR1] Bernard SA, Gray TW, Buist MD, Jones BM, Silvester W, Gutteridge G, Smith K (2002). Treatment of comatose survivors of out-of hospital cardiac arrest with induced hypothermia. N Eng J Med.

[CR2] Dzhala VI, Kuchibhotla KV, Glykys JC, Kahle KT, Swiercz WB, Feng G, Kuner T, Augustine GJ, Bacskai BJ, Staley KJ (2010). Progressive NKCC1-dependent neuronal chloride accumulation during neonatal seizures. J Neurosci.

[CR3] Eriksson K, Metsaranta P, Huhtala H, Auvinen A, Kuusela AL, Koivikko M (2005). Treatment delay and the risk of prolonged status epilepticus. Neurology.

[CR4] Glass HC, Wirrell E (2009). Controversies in neonatal seizure management. J Child Neurol.

[CR5] Glass HC, Glidden D, Jeremy RJ, Barkovich AJ, Ferriero DM, Miller SP (2009). Clinical neonatal seizures are independently associated with outcome in infants at risk for hypoxic-ischemic brain injury. J Pediatr.

[CR6] Gluckman PD, Wyatt JS, Azzopardi D, Ballard R, Edwards AD, Ferriero DM, Polin RA, Robertson CM, Thoresen M, Whitelaw A, Gunn AJ (2005). Selective head cooling with mild systemic hypothermia after neonatal encephalopathy: multicenter randomized trial. Lancet.

[CR7] Khot S, Tirschwell DL (2006). Long-term neurological complications after hypoxic-ischemic encephalopathy. Semin Neurol.

[CR8] Kleinman ME, de Caen AR, Chameides L, Atkins DL, Berg RA, Berg MD, Bhanji F, Biarent D, Bingham R, Coovadia AH, Hazinski MF, Hickey RW, Nadkarni VM, Reis AG, Rodriguez-Nunez A, Tibballs J, Zaritsky AL, Zideman D, Pediatric Basic and Advanced Life Support Chapter Collaborators (2010). Part 10: pediatric basic and advanced life support: 2010 International Consensus on Cardiopulmonary Resuscitation and Emergency Cardiovascular Care Science with Treatment Recommendations. Circulation.

[CR9] Krumholtz A, Stem BJ, Weiss HD (1998). Outcome from coma after cardiopulmonary resuscitation: Relation to seizures and myoclonus. Neurology.

[CR10] Rosen I (2006). The physiological basis for continuous electroencephalogram monitoring in the neonate. Clin Perinatol.

[CR11] Sessler DI (2001). Complications and treatment of mild hypothermia. Anesthesiology.

[CR12] Shankaran S, Laptook AR, Ehrenkranz RA, Tyson JE, McDonald SA, Donovan EF, Fanaroff AA, Poole WK, Wright LL, Higgins RD, Finer NN, Carlo WA, Duara S, Oh W, Cotton CM, Stevenson DK, Stoll BJ, Lemons JA, Guillet R, Jobe AH (2005). Whole-body hypothermia for neonates with hypoxic-ischemic encephalopathy. N Engl J Med.

[CR13] Spitzmiller RE, Phillips T, Meinzen-Derr J, Hoath SB (2007). Amplitude-integrated EEG is useful in predicting neurodevelopmental outcome in full-term infants with hypoxic-ischemic encephalopathy: a meta-analysis. J Child Neurol.

[CR14] van Rooij LG, Toet MC, Osredkar D, van Huffelen AC, Groenendaal F, de Vries LS (2005). Recovery of amplitude integrated electroencephalographic background patterns within 24 hours of perinatal asphyxia. Arch Dis Child Fetal Neonatal Ed.

[CR15] Young GB, Jordan KG, Doig GS (1996). An assessment of nonconvulsive seizures in the intensive care unit using continuous EEG monitoring: an investigation of variables associated with mortality. Neurology.

